# Adapting to evidence users: accessibility and incentives

**DOI:** 10.23889/ijpds.v9i5.2877

**Published:** 2024-09-18

**Authors:** Michael Lenczner

**Affiliations:** 1 Ajah

Adapting infrastructure for evidence creation and dissemination involves taking new technological, social, and environmental realities into account. Adaptations should also respond to what hasn’t changed: structural disincentives to evidence use among intended users. This presentation addresses the understudied problem of how administrative data systems go beyond enabling access to information – how data linkages or other infrastructure impacts policymakers or other intended information users. It addresses data sharing and data use, as well as public engagement and involvement.

A recent practitioner convening examined how new administrative data infrastructure can catalyze evidence-building, and, specifically explored the incentives and disincentives for stakeholders' use of evidence from evaluations. Participants discussed the multiple functions of evaluations for nonprofits, funders, policymakers, and communities, and shared lessons in capacity building from the recent history of nonprofit & human services evaluation. Preliminary results from this convening will be discussed in terms of their broader implications for the use of information resulting from future large-scale data linkages.

Broadly, the convening validated the hypothesis that efforts to increase the availability of evidence often proceed without sufficient consideration of the history of non-uptake of evidence, and that there has been limited acknowledgement of this problem by practitioners of evidence generation. Much work is needed within the evaluation & evidence, and nonprofit sector to address this challenge. Key themes for this work that emerged during the convening will illustrate what might work and what won’t work to address the non-uptake of evidence more broadly.

